# A consensus document on definition and diagnostic criteria for orthorexia nervosa

**DOI:** 10.1007/s40519-022-01512-5

**Published:** 2022-11-27

**Authors:** Lorenzo M. Donini, Juan Ramón Barrada, Friederike Barthels, Thomas M. Dunn, Camille Babeau, Anna Brytek-Matera, Hellas Cena, Silvia Cerolini, Hye-hyun Cho, Maria Coimbra, Massimo Cuzzolaro, Claudia Ferreira, Valeria Galfano, Maria G. Grammatikopoulou, Souheil Hallit, Linn Håman, Phillipa Hay, Masahito Jimbo, Clotilde Lasson, Eva-Carin Lindgren, Renee McGregor, Marianna Minnetti, Edoardo Mocini, Sahar Obeid, Crystal D. Oberle, Maria-Dolores Onieva-Zafra, Marie-Christine Opitz, María-Laura Parra-Fernández, Reinhard Pietrowsky, Natalija Plasonja, Eleonora Poggiogalle, Adrien Rigó, Rachel F. Rodgers, Maria Roncero, Carmina Saldaña, Cristina Segura-Garcia, Jessica Setnick, Ji-Yeon Shin, Grazia Spitoni, Jana Strahler, Nanette Stroebele-Benschop, Patrizia Todisco, Mariacarolina Vacca, Martina Valente, Màrta Varga, Andrea Zagaria, Hana Flynn Zickgraf, Rebecca C. Reynolds, Caterina Lombardo

**Affiliations:** 1 Sapienza University, Rome, Italy; 2 Universidad de Zaragoza, Zaragoza, Spain; 3 Heinrich Heine University Düsseldorf, Düsseldorf, Germany; 4 University of Northern Colorado, Greeley, USA; 5 Ecole de Psychologues Praticiens, Lyon, France; 6 University of Wroclaw, Wroclaw, Poland; 7 University of Pavia, Pavia, Italy; 8 Chung-Ang University, Seoul, South Korea; 9 University of Coimbra, CINEICC, Coimbra, Portugal; 10 University of Coimbra, Coimbra, Portugal; 11 University of Thessaly, Larissa, Greece; 12 Holy Spirit University of Kaslik, Jounieh, Lebanon; 13 Halmstad University, Halmstad, Sweden; 14 Western Sydney University, Sydney, Australia; 15 University of Michigan, Ann Arbor, USA; 16 University of Toulouse-Jean Jaurès, Toulouse, France; 17 The EN:SPIRE Clinic, Bengaluru, India; 18 Lebanese American University, Beirut, Lebanon; 19 Texas State University, San Marcos, USA; 20 University of Castilla-La Mancha, Ciudad Real, Spain; 21 The University of Edinburgh, Edinburgh, UK; 22 University of Bordeaux, Bordeaux, France; 23 Institute of Psychology, ELTE Eötvös Loránd University, Budapest, Hungary; 24 Northeastern University, Boston, USA; 25 University of Valencia, Valencia, Spain; 26 Universitat de Barcelona, Barcelona, Spain; 27 University of Catanzaro, Catanzaro, Italy; 28 International Federation of Eating Disorder Dietitians, Dallas, USA; 29 Chung-Ang University, Seoul, South Korea; 30 University of Freiburg, Freiburg, Germany; 31 University of Hohenheim, Stuttgart, Germany; 32 Casa Di Cura Villa Margherita, Vicenza, Italy; 33 Amedeo Avogadro University of Eastern Piedmont, Vercelli, Italy; 34 Semmelweis University, Budapest, Hungary; 35 Children’s Healthcare of Atlanta, Emory University, Atlanta, USA; 36 ICS MAUGERI IRCCS, Pavia, Italy; 37 Effat University (KSA), Jeddah, Saudi Arabia; 38 Lapeyronie Hospital, CHRU Montpellier, Montpellier, France; 39 School of Population Health, UNSW Sydney, Sydney, NSW 2052 Australia

**Keywords:** Orthorexia nervosa (ON), Feeding and eating disorders (F&ED), Anorexia nervosa (AN), Obsessive–compulsive disorder (OCD), Avoidant restrictive food intake disorder (ARFID), Eating disorder

## Abstract

**Purpose:**

Since the term orthorexia nervosa (ON) was coined from the Greek (ὀρθός, right and ὄρεξις, appetite) in 1997 to describe an obsession with “correct” eating, it has been used worldwide without a consistent definition. Although multiple authors have proposed diagnostic criteria, and many theoretical papers have been published, no consensus definition of ON exists, empirical primary evidence is limited, and ON is not a standardized diagnosis. These gaps prevent research to identify risk and protective factors, pathophysiology, functional consequences, and evidence-based therapeutic treatments. The aims of the current study are to categorize the common observations and presentations of ON pathology among experts in the eating disorder field, propose tentative diagnostic criteria, and consider which DSM chapter and category would be most appropriate for ON should it be included.

**Methods:**

47 eating disorder researchers and multidisciplinary treatment specialists from 14 different countries across four continents completed a three-phase modified Delphi process, with 75% agreement determined as the threshold for a statement to be included in the final consensus document. In phase I, participants were asked via online survey to agree or disagree with 67 statements about ON in four categories: A–Definition, Clinical Aspects, Duration; B–Consequences; C–Onset; D–Exclusion Criteria, and comment on their rationale. Responses were used to modify the statements which were then provided to the same participants for phase II, a second round of feedback, again in online survey form. Responses to phase II were used to modify and improve the statements for phase III, in which statements that met the predetermined 75% of agreement threshold were provided for review and commentary by all participants.

**Results:**

27 statements met or exceeded the consensus threshold and were compiled into proposed diagnostic criteria for ON.

**Conclusions:**

This is the first time a standardized definition of ON has been developed from a worldwide, multidisciplinary cohort of experts. It represents a summary of observations, clinical expertise, and research findings from a wide base of knowledge. It may be used as a base for diagnosis, treatment protocols, and further research to answer the open questions that remain, particularly the functional consequences of ON and how it might be prevented or identified and intervened upon in its early stages. Although the participants encompass many countries and disciplines, further research will be needed to determine if these diagnostic criteria are applicable to the experience of ON in geographic areas not represented in the current expert panel.

**Level of evidence:**

Level V: opinions of expert committees

**Supplementary Information:**

The online version contains supplementary material available at 10.1007/s40519-022-01512-5.

## Introduction

Orthorexia nervosa (ON) was first described by family doctor Steven Bratman in 1997, using a neologism coined from the Greek (ὀρθός, right and ὄρεξις, appetite) to describe a fixation on “correct” eating he had observed among his patients. In 2016, Bratman and Dunn differentiated ON from a general desire for a healthy lifestyle by specifying that it causes negative consequences such as malnutrition and/or social functioning impairment [1].

ON, often truncated to “orthorexia,” appears worldwide in both scientific literature and common usage to describe an overvaluation and preoccupation with food quality and its impact on health, but never with a consistent definition or standardized diagnostic criteria. ON is not recognized in the most recent Diagnostic and Statistical Manual of Mental Disorders (DSM-5-TR) or International Classification of Diseases (ICD-11), and there is some debate whether ON is a distinct mental disorder at all [2–6].

Although several authors have proposed diagnostic criteria for ON (see [7] for a review), there is no standard or consensus. This leaves the functional definition of ON up to individual researchers or authors, with resulting confusion and research findings that are not generalizable. For example, food habits characterizing ON have been described in the literature as “restrictive,” “ritualized,” “strictly controlled” and “distorted,” among others—all non-measurable terms. When describing the nature of foods for purposes of determining if an individual has ON, they may be called “healthy,” “correct,” “organic,” “pure,” “nutritious,” “acceptable,” or “safe.” Those are just two samples of the wide variation in terminology. Without a standardized diagnosis or a shared definition of ON in the scientific literature, readers cannot compare one study with another, and research cannot be conducted to identify risk and protective factors, pathophysiology, functional consequences, or evidence-based therapeutic approaches.

A separate but related dilemma is that no empirical data to date show that ON is a distinct entity from other disorders with overlapping features. Without a standard definition, ON is in limbo, and we are unable to answer the question of whether it should be considered a distinct condition for purposes of diagnosis and treatment, or as a subset of another disorder with overlapping features, such as anorexia nervosa (AN), avoidant-restrictive food intake disorder (ARFID), or obsessive–compulsive disorder (OCD) [8–16]. This gap is most prominently illustrated by the utter lack of studies focusing on prospective treatments for ON. The current study is not a theoretical exercise. Consolidating the current knowledge about ON from researchers and professionals in practice into a consensus definition is the foundation required for interdisciplinary collaboration, evaluation and revision of existing tools or development of new tools for investigation of prevalence, better identification and screening, development and refinement of treatment protocols, and appropriate care of individuals. We know that many individuals worldwide are experiencing the negative effects of ON. Adoption of consistent terminology and defining characteristics for ON – either on its own or as a feature of another disorder—is the first step toward appropriate care [10, 17–19].

## Methods

In November 2020, a worldwide grouping of researchers and treating professionals was invited by a steering committee to pool their knowledge and clinical experience about ON. Invitation criteria was having one or more published works related to ON.

The purposes of the consensus-building process were described as follows: (1) Reach a shared definition of ON and propose tentative diagnostic criteria; (2) Evaluate in which DSM chapter and category ON should be included; (3) Describe, if possible, the course, risk and protective factors for ON, medical and psychiatric comorbidity, differential diagnosis, and psychological and functional consequences.

**Fig. 1:**
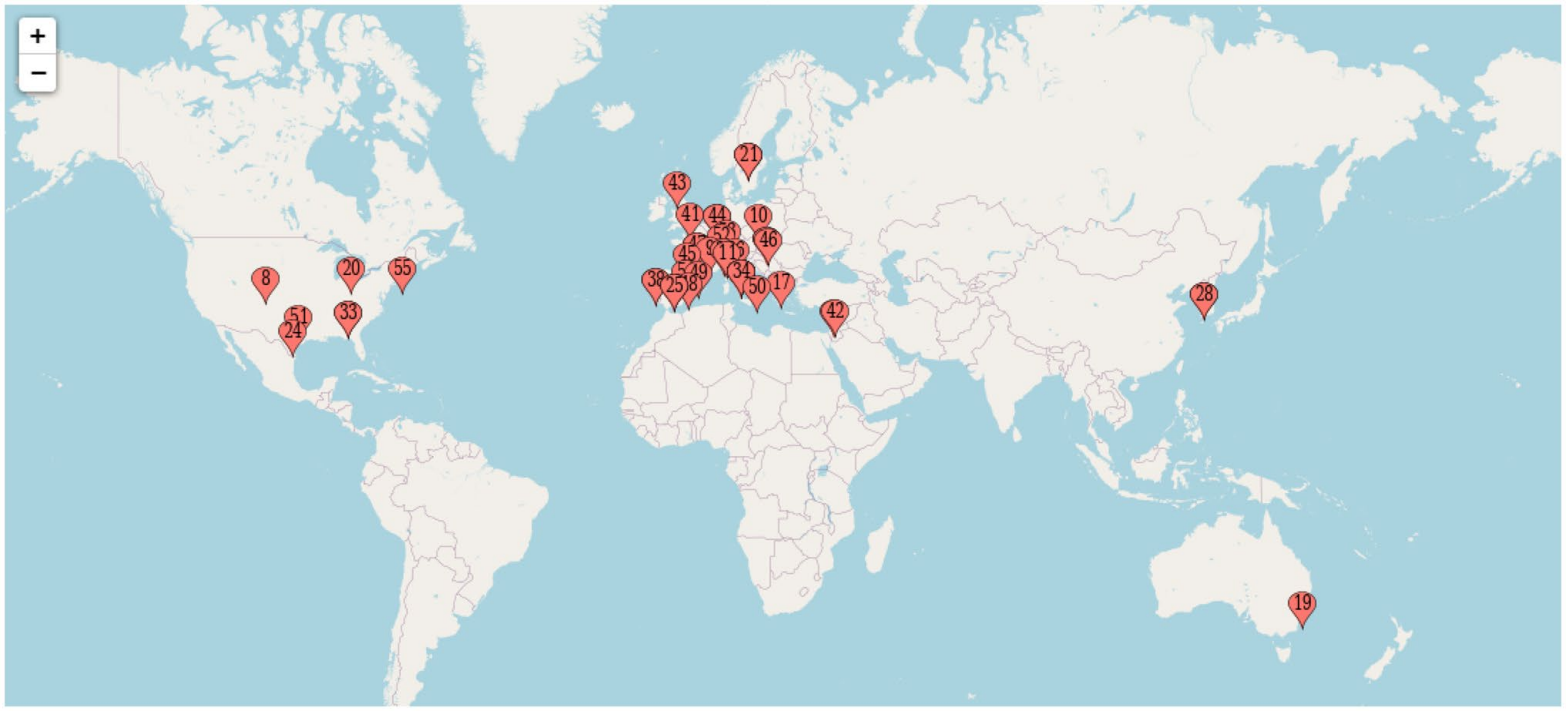
Geographic distribution of the contributors

47 of the invited experts, representing 14 countries across four continents, participated in the process and are listed as co-authors of this paper. Figure 1 shows the geographic distribution of the participants. Five members of the group (JRB, FB, LMD, TMD, CL) constituted the steering committee that discussed results and considered the comments of the panel of experts, and two members of the steering committee (LMD and CL) coordinated and supervised the study activities.

For phase I of the Delphi process, the steering committee used the standard operating procedures for ESPEN guidelines and consensus papers to identify 67 statements in five categories related to ON (Table S1) [20, 21]. The categories were the following: A–Definition, Clinical Aspects, Duration; B–Consequences; C–Onset; D–Exclusion Criteria; Other Characteristics. Table 1 shows the number of statements in each category in each phase. All 67 statements were submitted to the expert panel via an email attachment. Participants were asked to choose whether they agreed or disagreed with each statement, and to offer comments and rationale for their choice.

**Table 1:** Number of statements proposed in the three phases of the process of the consensus definition

Statements proposed in …	First round	Second round	Final statements selected
Criterion A: Definition and clinical aspects	15	9	7
Duration	2	1	1
Criterion B: Consequences	9	4	3
Criterion C: Onset	2	1	2
Criterion D: Exclusion criteria	3	3	3
Other characteristics associated or possibly risk factors	26	13	9
Differential diagnosis with other psychiatric diseases	10	3	3

After reviewing all responses, incorporating written comments from participants, and in accordance with the Delphi procedure, the steering committee passed through to phase II the 34 statements that met or exceeded the predetermined consensus threshold of 75% (Table S2). Once again, participants were asked to choose whether they agreed or disagreed with each statement, and to offer comments and rationale for their choice.

After phase II, 27 statements met or exceeded the 75% threshold (Table 2). These 27 statements and their corresponding comments from participants were included in phase III, where participants were once again asked if they agreed or disagreed and invited to provide rationale, as well as being asked to answer open-ended questions and suggest a future research agenda. The responses to this final phase are presented below.

**Table 2:** Statements proposed and agreement from the expert panel

CRITERION A: DEFINITION, CLINICAL ASPECTS AND DURATION		
A1. Definition		
1	Orthorexia Nervosa (ON) is a mental health disorder associated with reduced wellbeing and falling within the DSM-5 category of “Feeding and Eating Disorders” (F&ED)	Agreement (%) * 93.3
2	The definition of “healthful eating” or “pure eating” includes a dietary theory or set of beliefs whose specific details may vary. Subjects with ON often refer to “healthy" food as pure, clean, organic, right, correct, natural, safe; “unhealthy” food is often referred to as processed, with added ingredients, prepared, treated, toxic, contaminated such as to represent harmful consequences for the individual’s health. It might also include any other definition of healthy or unhealthy according to the affected individual (his/her background/culture/knowledge/moment in life) or to dietary trends and cultures	96.7
3	ON is characterized by a strong preoccupation with one's eating behavior and with self-imposed rigid and inflexible rules which are strictly controlled and include spending an excessive amount of time for planning, obtaining, preparing and/or eating one’s food	93.3
4	ON-related behavior involves disturbances of eating habits that lead to a nutritionally unbalanced diet, that negatively affects health status (both physical and mental health), and quality of life	93.3
A2. Aspects that are frequently present in subjects with ON		
5	ON includes emotional (e.g. feeling guilty after having eating food considered to be unhealthy), cognitive (e.g. problems concerning attention and concentration) and/or social (e.g. social exclusion) consequences, that have a negative effect on the individuals educational, work or social life	96.6
6	In particular individuals with ON experience emotional distress, anxiety (if they are confronted with food they believe to be unhealthy and they fear they might be impaired by eating them), problems concerning attention and concentration (if an individual thinks about healthy eating all day) and a feeling of guilt as a consequence of not being able to eat healthy	96.6
7	In ON the adherence to self-imposed dietary rules has an undue influence on self-esteem	90
A3. Duration		
8	Symptoms should be present during the last 6 months. However, if there is a severe impairment of health (e.g. severe malnutrition), the diagnosis can be given even after 3 months	78.6
CRITERION B: CONSEQUENCES		
9	As a result of the excessive amount of time devoted to their diet (reading about, acquiring and/or preparing foods), ON has a negative impact on other important areas of psychosocial and personal functioning	96.7
10	The food selectivity, that characterizes ON individuals, can contribute to cause nutritional deficiencies (e.g. anemia, extreme weight loss, global or selective malnutrition) and hormonal disturbances	96.4
11	The rigid eating rules may result in low body weight and sometimes the sociocultural ideals of healthiness, at least in Western countries, may overlap greatly with thin and muscular ideals. However, this low weight may be better conceived as a side effect or a consequence of ON instead of as the result of body dissatisfaction	85.2
CRITERION C: ONSET of ON		
12	ON seems to be associated with the development of other forms of EDs and/or with migration to other forms of F&EDs. It may precede others F&EDs, coexist with EDs (when orthorexic attitude represents a more socially acceptable way of deploying the F&ED), follow other F&EDs (representing in this case a faulty coping strategy when no longer able to practice other F&ED behaviors). It might serve as a coping strategy for individuals affected with AN to continue restricting their diet	87.3
13	People may develop ON as a consequence of prescribed or self-prescribed dietary rules related or not to clinical conditions (e.g. people with chronic/somatic diseases requiring specific restrictions; seeking food theories to adhere to cure a chronic disease such as fibromyalgia, food allergies or intolerances). ON may also represent in these cases a coping mechanism of chronic diseases in which strict dieting is needed (feeling of control)	87,3
CRITERION D: EXCLUSION CRITERIA		
14	The food selection and/or exclusion from the diet is not attributable to a clinical dietary prescription (e.g. in renal insufficiency, obesity, food allergies and intolerances)	79.3
15	If clinical conditions are present and motivate food selection and/or exclusion, the onset of ON is characterized by a food selection and/or exclusion that is excessive, inappropriate, and goes beyond standard medical advice and practices	79.3
16	The food selection and/or exclusion from the diet is not attributable to economic conditions, values, cultural, religious beliefs or delirious ideas	96.7
OTHER CHARACTERISTICS ASSOCIATED OR POSSIBLY RISK FACTORS		
17	Competitive sports, athletic performance concerns and high physical exercise frequency	75.9
18	History of others F&EDs or mental disorders (e.g. OCD)	93.1
19	(Psycho) somatic problems, hypochondria, depressive symptoms, anxiety (generalized or specific)	89.3
20	Perfectionism, need of control, low self-esteem, narcissism, self-criticism and tendency to impose excessively high standards for oneself	92.9
21	Being excessively influenced by media, social networks, online platforms and websites related to eating behaviors and/or physical appearance	83.3
22	Vegan, vegetarian eating habits	90
23	Emotion dysregulation	86.2
24	University and professional choices (e.g. dietician, nutritionist)	88
DIFFERENTIAL DIAGNOSIS with other psychiatric diseases		
25	The fundamental differences with anorexia nervosa (AN) are that: In ON, appearance concerns are not central, physical appearance is not overvalued and there is no explicit/aware search for thinness In AN, the goal is to lose weight/ maintain current weight while in ON the main goal is to be as healthy as possible In AN, self-esteem revolves around weight/shape while In ON self-esteem revolves around the ability to follow the self-imposed dietary rules 'Weight/shape phobia' in a person with ON, if present is an 'implicit attitude'	92.6
26	The fundamental differences with obsessive compulsive disorders (OCD) are that In ON, obsessions (overvalued ideas) and compulsions only concern eating behavior and health Individuals with OCD experience ego-dystonic obsessions and try to ignore or suppress those unwanted thoughts and urges, whereas individuals with ON experience ego-syntonic obsessions about food/eating that are considered appropriate and that they do not want to ignore	93.1
27	The fundamental differences with the avoidant-restrictive food intake disorder (ARFID) are that in these individuals - The diagnostic marker is malnutrition and low body weight due to food restriction as a consequence of: - An aversive experience with food causing a conditioned negative response to eating, such as choking - Apparent lack of interest in eating - Highly selective eating based on the sensory properties of food, such as color, taste, or texture On the contrary, in ON food restriction is the result of worries about the healthiness of a certain food and malnutrition may represent a consequence and not a diagnostic marker Patients with ARFID are afraid about consequences on the very short term (e.g., vomiting, choke, …) while patients with ON are afraid about consequences on the long term (e.g., diabetes, cancer, high cholesterol)	86.2

^*^% of agreement at the end of the second round

## Results

The results of this process are the first published consensus-built proposed criteria for orthorexia nervosa. They are presented here in their entirety, along with comments, open questions, and directions for future research. Numbers in parentheses signify the percentage of the expert panel who agreed on each statement.

### Criterion A: definition, clinical aspects and duration

#### A1. Definition

1. ON is characterized by a strong preoccupation with one's eating behavior and with self-imposed rigid and inflexible rules which are strictly controlled and include spending an excessive amount of time for planning, obtaining, preparing and/or eating one’s food (agreement 93.3%).
2. The definition of “healthful eating” or “pure eating” includes a dietary theory or set of beliefs whose specific details may vary. Subjects with ON often refer to “healthy" food as pure, clean, organic, right, correct, natural, safe; “unhealthy” food is often referred to as processed, with added ingredients, prepared, treated, toxic, contaminated such as to represent harmful consequences for the individual’s health. It might also include any other definition of healthy or unhealthy according to the affected individual (his/her background/culture/knowledge/moment in life) or to dietary trends and cultures (agreement 96.7%).
3. Orthorexia Nervosa (ON) is a mental health disorder associated with reduced wellbeing and falling within the DSM-5 category of “Feeding and Eating Disorders” (agreement 93.3%).
4. Individuals with ON experience emotional distress, anxiety (if they are confronted with food they believe to be unhealthy and they fear they might be impaired by eating them), problems concerning attention and concentration (if an individual thinks about healthy eating all day) and a feeling of guilt as a consequence of not being able to eat healthy (agreement 96.6%).
5. In ON, the adherence to self-imposed dietary rules has an undue influence on self-evaluation (agreement 90%).

##### Comments and open questions for future research

- ON appears to be more closely related to F&ED than to OCD although obsessive–compulsive characteristics are present [9].
- The adoption of “healthful eating” or “pure eating” (as defined in Criterion A1 statement 2) is necessary but not sufficient for a diagnosis of ON since actions toward these goals are highly variable and not always harmful. Additionally the terms "healthy" and "unhealthy" can be subjectively defined very differently, leaving room for personal interpretations of these terms. For example, definitions of “healthy” sometimes include low-calorie/low-fat/low-sugar foods and dietary restrictions that may be similar to those of weight-loss dieters or people with weight/shape concerns and/or F&ED. This aspect is further conditioned by aspects of the current or local culture that may affect the attitude toward healthy/orthorexic behaviors and perceived quality of life [22].
- Research has shown individuals with ON report eating (and other lifestyle) behavior that is relatively unhealthy suggesting the existence of individuals with high levels of ON and unhealthy lifestyles [2, 23, 24].
- People affected by ON, and more generally people affected by mental disorders, frequently have an ambivalent attitude toward their own disorder. Sometimes they agree that their behavior is accompanied by distress, but sometimes they are not fully aware of the impairment and distress their behaviors and attitudes entail [25].
- ON may affect physical health status similarly to other mental disorders (e.g., depression, specific phobia, and OCD) that do not include physical impairment as a criterion. However, the panel considers the impact of ON on health status differently from other mental disorders, since it is a fundamental aspect of the disease (as in F&ED) and not just one of many possible consequences.
- The specific behavioral characteristics of ON indicated in the criteria statements (e.g., “spending an excessive amount of time for are still subject to discussion and research by the expert panel.
- Empirical support for the presence/level of emotional distress associated to ON is mixed [26–28].
- Recent proposals have been made to distinguish between ON as defined here and “healthy orthorexia” that refers to a non-pathological interest in healthy eating and nutrition [29–31].
- Often individuals with ON do not complain of the ON symptoms themselves, but of the consequences of transgressing the strict self-imposed rules and dietary restrictions [25].
- Poor insight into illness is a typical characteristic of ON, and more in general of F&ED, especially toward the onset/early stages of the disorder [25].
- The presence of overvalued ideas about food and their consequences (e.g., a specific food causes a specific disease) unduly influences eating attitudes and behavior and self-evaluation.

#### A2. Duration

6. Symptoms should be present for at least the last 6 months. However, if there is a severe impairment of health (see Criterion B10) or psychosocial functioning, the diagnosis can be given after only 3 months (agreement 78.6%).

##### Comments and open questions for future research

The panel agrees that this duration criterion, although arbitrary at the moment, is necessary and commonly present in the definition of most mental disorders. More research is needed to properly identify a more meaningful temporal benchmark.

### CRITERION B: CONSEQUENCES

7. ON-related behavior involves disturbances of eating habits that lead to a nutritionally unbalanced diet that negatively affects health status (both physical and mental health), and quality of life (agreement 93.3%)
8. ON includes emotional (e.g., feeling guilty after having eating food considered to be unhealthy), cognitive (e.g., problems concerning attention and concentration) and/or social (e.g., social exclusion) consequences, that have a negative effect on the individual’s educational, work or social life (agreement 96.6%).
9. As a result of the excessive amount of time devoted to their diet (reading about, acquiring and/or preparing food), ON has a negative impact on other important areas of psychosocial and personal functioning (agreement 96.7%).
10. The food selectivity that characterizes ON individuals, can contribute to cause nutritional deficiencies (e.g., anemia, extreme weight loss, global or selective malnutrition) and hormonal disturbances (agreement 96.4%).
11. The rigid eating rules present in ON may result in low body weight that corresponds to sociocultural ideals of healthiness, at least in Western countries, or may overlap greatly with thin and muscular ideals. However, this low weight may be better conceived as a side effect or a consequence of ON instead of as the result of body dissatisfaction (agreement 85.2%).

#### Comments and open questions for future research

- The panel agrees that consequences of ON need to be further investigated. Functional impairment has not been examined extensively [6]. In the available case reports and prevalence surveys conducted in non-clinical samples, the impact of orthorexic symptoms on general psychosocial functioning have been shown to be significant but with small effect sizes [26, 32]. Future studies are warranted to disentangle which specific psychosocial domains (e.g. health status, emotional distress, family financial management, relationships, etc.), are affected by ON symptoms.
- Nutritional consequences need to be evaluated considering the sociocultural ideals (e.g., prevailing eating models/myths and esthetic models) that may lead people to change their diet as well as their personal definition of health.
- A current tendency in western countries is to equate health with weight/body appearance (and therefore body dissatisfaction), and healthy food with a way of losing weight or modifying appearance. The diet industry encourages this using vocabulary such as health/healthy/wellness/wellbeing to promote their products.
- The panel considers that if an individual’s drive to lose weight is associated with health beliefs and/or anxiety, then it is consistent with ON. If the drive to lose weight is associated with body dissatisfaction, dysphoria, and/or dysmorphia, then it may be more consistent with AN. A recent review found that across studies, ON symptoms were consistently and moderately-to-strongly related to measures of restrained eating (e.g., dieting) and drive for thinness, whereas relationships between ON and measures of body image ranged widely from negligible to moderate [8]. The boundaries between AN and ON are not clear enough, and further research is needed on the significance of and motivations behind weight-loss behaviors in ON.
- Although 85% of the panel agree that rigid eating rules may result in low body weight, research has been inconclusive on the relationship between ON and body mass index (BMI). Studies have shown positive correlation, negative correlation, or no relationship. A poorly chosen dietary intake based on ON-related incorrect nutritional theories may result in weight loss or gain [33–35]. In this regards, further work needs to be conducted to ascertain whether a BMI threshold does exist for distinguishing weather extreme underweight in ON becomes indicative of underlying anorexic tendencies.
- Individuals experiencing ON may impose inflexible, inappropriate and even deadly subsets of their own dietary rules on infants, toddlers, or young children in their care (ON per proxy) [36].
- Future research should include and examine severity specifiers to (a) meet the need of the international diagnostic system, (b) refine the diagnostic process and (c) develop improved treatment interventions.

### Criterion C: ONSET of ON

12. ON seems to be associated with the development of other forms of F&EDs and/or with migration to other forms of F&EDs. It may precede other F&EDs, coexist with F&EDs (when an orthorexic attitude represents a more socially acceptable way of deploying the F&ED), follow other F&EDs (representing a faulty coping strategy when no longer able to practice other F&ED behaviors), or serve as a coping strategy for individuals affected with AN to continue restricting their diet (agreement 87.3%).

13. People may develop ON as a consequence of prescribed or self-prescribed dietary rules related or not to clinical conditions (e.g., people with chronic/somatic diseases requiring specific restrictions; seeking food theories to adhere to cure a chronic disease such as fibromyalgia, food allergies or intolerances). ON may also represent in these cases a coping mechanism of chronic diseases in which strict dieting is needed (feeling of control) (agreement 87.3%).

#### Comments and open questions for future research

- The co-existence of ON and other F&Eds, upon which the panel agreed (as indicated by the 87.3% of agreement for statement 12) may appear inconsistent with the DSM assumption of a hierarchical position of mutual exclusion among the F&Eds. However this co-existence may be an exception to this rule (like for the pica disorder) or the statement should be changed in the future by new research findings.
- Social media, fitness influencers, health professionals not specialized in eating disorders or nutrition may contribute to the onset of ON by sharing, spreading, or prescribing food rules and incorrect dietary theories as well as their personal convictions about weight and eating. Individuals at risk for development of ON may experience confusion or distress in their attempts to follow conflicting guidance [37, 38].
- More research is needed on links between ON onset and other non F&ED mental disorders such as OCD, anxiety, post-traumatic stress disorder, as well as which individuals are at risk of developing ON as a result of authority-prescribed or self-prescribed dietary rules [12].

### Criterion D: exclusion criteria

- 14. The food selection and/or exclusion from the diet is not attributable to a clinical dietary prescription (e.g., in renal insufficiency, obesity, food allergies and intolerances) (agreement 79.3%).
- 15. If clinical conditions are present and motivate food selection and/or exclusion, the onset of ON is characterized by a food selection and/or exclusion that is excessive, inappropriate, and goes beyond standard medical advice and practices (agreement 79.3%).
- 16. The food selection and/or exclusion from the diet is not attributable to economic conditions, values, cultural, religious beliefs or delusional ideas (agreement 96.7%)

#### Comments and open questions for future research

- The panel considers that food selection/exclusion in ON cannot be attributable to cultural or religious beliefs, but the role of these aspects needs to be better analyzed. It is unclear whether certain socially-reinforced food behaviors, for example a governmentally-enacted sugar tax, might play a role in ON development or lead to new cultural norms that would encourage ON behaviors.
- Some psychiatric comorbid disorders (e.g., OCD, somatoform disorders, delusional disorder) may share symptoms with ON. A differential diagnosis is therefore mandatory to avoid underdiagnosis/misdiagnosis of ON.

#### OTHER CHARACTERISTICS ASSOCIATED AND/OR POSSIBLE RISK FACTORS

Although evidence is not always consistent, the panel agrees that ON seems to be associated with:

17. Competitive sports, athletic performance concerns and high physical exercise frequency (agreement 75.9%)
18. History of other F&EDs or mental disorders (e.g., OCD) (agreement 93.1%)
19. (Psycho)-somatic problems, hypochondria, depressive symptoms, anxiety (generalized or specific) (agreement 89.3%)
20. Perfectionism, need of control, low self-evaluation, narcissism, self-criticism and tendency to impose excessively high standards for oneself (agreement 92.9%)
21. Being excessively influenced by media, social networks, online platforms and websites related to eating behaviors and/or physical appearance (agreement 83.3%)
22. Vegan, vegetarian eating habits (agreement 90%)
23. Emotion dysregulation (agreement 86.2%)
24. University and professional choices (e.g., dietician, nutritionist) (agreement 88%)

#### Comments and open questions for future research

- All characteristics associated with ON in this section need further research to enhance consistency of findings and to better understand their correlation with ON.
- In particular, the panel did not reach the consensus threshold on the association of ON with “higher level of education and sociocultural status,” “specific age and gender,” “body weight concerns or variations during life,” “physical shape or body image disturbances,” “impulsivity and appearance anxiety,” or “alcohol and drug addiction.” Existing research on these factors is insufficient and inconclusive [2, 19, 24, 34, 39–41].
- Although the panel agrees that intense/frequent physical exercise related to competitive sports and performance concerns may be associated with ON, further research is needed on performance-related dietary advice given to athletes, the role of a trainer or coach’s dietary instruction/attitude, and the general social environment toward eating on the development of ON. Research is needed to verify the potential impact of a career-ending injury or inability to exercise on the pathogenesis of ON due to the belief that without exercise, certain foods are unacceptable.
- The association of ON with vegan and vegetarian diets is based on motives regarding health, esthetics and healing rather than motives regarding animal welfare and ethical aspects [42, 43]. Although a large number of investigations have found that vegetarian and vegan diets are associated with greater orthorexic tendencies hypothesizing their role as possible risk factors in developing ON [see 2 for an extensive discussion], it is worth noting that veganism and vegetarianism involve the practice of abstaining from all products derived at least partly from animals, promoting the consumption of animal-free alternatives to avoid all forms of exploitation and cruelty to animals. Thus vegetarian and vegan diets should not be confused with a pathological obsession with healthy eating due to overvalued ideas regarding the health benefits of food.
- The contribution of social media apps and sites to development of ON may be deeply influenced by the an individual user’s level of media competence and their pre-existing, underlying mental status, in addition to the social media content and algorithms. Some people participate heavily in social media and don’t exhibit ON. The question is rather what people are looking for when they use social media (e.g., validation, a way to feel part of a community) and if they find it in dieting/orthorexic encouragement.
- The higher prevalence of F&ED and ON in health students and professionals as demonstrated in the literature may be associated with previous or concurrent F&ED guiding their university or professional choices. However, some studies also showed that ON tendencies decreased in health students during the course of their studies [44].

#### DIFFERENTIAL DIAGNOSIS with other psychiatric diseases (ON vs AN)

25. The fundamental differences between ON and AN are that (agreement 92.6%):
• In ON, appearance concerns are not central, physical appearance is not overvalued and there is no explicit/aware search for thinness
• In AN, the goal is to lose weight/ maintain current weight while in ON the main goal is to be as healthy as possible
• In AN, self-evaluation revolves around weight/shape while in ON self-evaluation revolves around the ability to follow the self-imposed dietary rules to improve health status/avoid negative consequences for health
• 'Weight/shape phobia' in a person with ON, if present, is an 'implicit attitude', namely the person is not aware of it.

#### Comments and open questions for future research

- Individuals with ON may display implicit or explicit body image distortion [45]. Further research is needed to determine similarities and differences in body image issues across the eating disorder spectrum using a transdiagnostic model.
- Further research is needed to better define the role of physical appearance concerns in ON, including negative body image, body dissatisfaction, dysphoria and dysmorphia, overvaluation of weight and shape in self-valuation, the diet industry, evolving Western societal influences, and health justification as a pretext to control eating.

#### DIFFERENTIAL DIAGNOSIS with other psychiatric diseases (ON vs OCD)

26. The﻿ fundamental differences between ON and OCD are that (agreement 93.1%):
• In ON, obsessions (overvalued ideas) and compulsions only concern eating behavior and health.
• Individuals with OCD experience ego-dystonic obsessions and try to ignore or suppress those unwanted thoughts and urges, whereas individuals with ON experience ego-syntonic obsessions about food/eating that are considered appropriate and desirable, that they do not want to ignore.

#### Comments and open questions for future research

• The﻿ content of obsessions and compulsions may vary among individuals with OCD, although there are common themes such as: cleanliness, symmetry, forbidden thoughts or taboos, harm [3]. In contrast, in ON the content is mostly limited to the theme of food and health.
• ON and OCD share cognitive rigidity, perfectionism traits, obsessions, and compulsions. However, in ON all these aspects focused to the domain of healthy food, including obsessions and ritualistic behavior related to meal purchase, preparation, and consumption which are perceived as normal and adequate. Moreover, OCD obsessions are usually perceived as ego-dystonic (i.e. they are experienced as strangers to themselves) and are often associated with severe distress and desire to change [9].

#### DIFFERENTIAL DIAGNOSIS with other psychiatric diseases (ON vs ARFID)

27. The﻿ fundamental differences between ON and ARFID are that (agreement 86.2%):
• In ARFID, the diagnostic markers are malnutrition, low body weight and psychosocial impairment due to food restriction as a consequence of an aversive experience with food causing a conditioned negative response to eating (such as choking), apparent lack of interest in eating, or highly selective eating based on the sensory properties of food, such as color, taste, or texture.
• On the contrary, in ON food restriction is the result of worries about the healthiness of a certain food and malnutrition may represent a consequence and not a diagnostic marker.
• Patients with ARFID are afraid of consequences on the very short term (e.g., vomiting, choking) while patients with ON are afraid of consequences on the long term (e.g., diabetes, cancer, high cholesterol).

#### Comments and open questions for future research

- Research should clarify if ON could be considered a fourth subtype of ARFID or should be included under the third subtype of ARFID to avoid the continued multiplication of diagnostic categories.
- ON can occur as a result of a negative/traumatic event, either food-related or not. ON cannot be ruled out if someone has had a choking episode or eliminates food based on perceived health. If subjects avoid the food that caused choking or all foods because of a fear of choking, they can be classified more adequately as experiencing ARFID, but if the perceived healthiness/purity of foods plays a role, then ON may be more appropriate.
- Food restriction in ARFID is associated with beliefs about the immediate consequences of eating or motivational factors related to food and/or specific eating events, whereas ON (like AN) involves food restriction associated with longer-term consequences of eating.

## Discussion

This paper addressed an important gap in our understanding of the constellation of behaviors described under the umbrella term “Orthorexia Nervosa”—namely that despite increasing research, ON is not consistently defined and has not received its own classification in any standardized categorization system [3]. This is partially due to an ongoing debate regarding its validity as a disorder distinct from other already-defined mental conditions and hinders the development of screening and treatment protocols.

As evidenced by mounting interest in orthorexia research and individuals presenting to treatment with orthorexia symptoms, both patients and health professionals would benefit from a better understanding of ON, possible only with establishment of preliminary diagnostic criteria.

The 27 proposed criteria that resulted from the current investigation reflect high agreement among the international, multidisciplinary expert panel about ON and its related risk factors, pathophysiology, clinical, psychological and functional consequences. This work represents a crucial starting point for future studies of ON and hopefully will overcome some of the skepticism that at times accompanies discussions of ON as a mental disorder rather than a “lifestyle choice.”

All 27 criteria included had high agreement from the panel, from 75.9% to 96.7%, providing a validated starting point for future ON research and a foundation for continued refinement of the diagnosis. The domains with the most divergence were the duration (78.6%) and exclusion criteria (79.3%), whereas the panel overall strongly agreed on the other statements (from 83.3 to 96.7% of agreement). Particularly high agreement (above 90%) was reached on all Criterion A statements except A3 (duration), demonstrating that this panel clearly believes that ON is a distinct disorder associated with probably impaired heath status and reduced wellbeing, comparable other F&ED. These conclusions are in line with recent meta-analytic findings indicating ON symptoms were more associated with F&EDs than OCD, but that pooled effect size for the relationship with F&ED were in the moderate range, with a significant amount of non-overlapping variance between the constructs, and thus could be treated as a stand-alone form of eating disorder [9].

As regards the relationship with OCD, the pooled association reported by Zagaria and colleagues [9] is small. Moreover, consistently with previous evidence it suggests that ego-syntonic content of obsessions characterizes individuals with high ON symptoms differently from the ego-dystonic obsessions found in OCD.

As in OCD, individuals with ON may or may not be aware of their disorder and/or of the consequences of the disorder on their health and wellbeing [46]. However, in OCD only some individuals has a poor insight into the correctness of beliefs underlying their obsessive–compulsive symptoms and only few (less than 4%) has no insight [3]. This specifier should be evaluated by future studies addressing the degree of insight into the correctness of beliefs underlying ON symptoms, since it may be vital for the outcome. The individual’s awareness of ON disorder and its consequences may be partially due to the limited nutritional knowledge associated with ON, including irrational and incorrect beliefs on “healthy eating” [44], as well as the social desirability of “healthy eating” [11]. As an example, it was observed elevated endorsement of ON cognitions and behaviors was associated with unhealthy eating behavior according to recognized nutrition standards and reported low nutrition literacy [8, 30], suggesting that for some this disorder may stem from lack of knowledge similarly to what happens in other ED [47]. Moreover, clinicians have to be very cautious in thinking that the thoughts of those suffering from ON are always obsessive ideas. Often subjects suffering from ON report mainly persistent and excessive ideas or phobias with respect to healthy food, rather than real intrusive, ego-dystonic obsessions. Therefore, particular attention must be paid to evaluating the difference between obsession, rigid and pre-dominant ideas and phobias in subjects with ON.

Criterion B reflects the short- and long-term consequences of ON in psychosocial and personal functioning as well as in nutritional and weight status. A crucial point concerned the ambiguous relationship between ON and psychological functioning. Literature suggests mixed evidence on the association between ON symptoms and several maladaptive psychological characteristics such as perfectionism [26, 28], body dissatisfaction [8, 48, 49], and self-evaluation [28, 34]. Such aspects seem particularly relevant insofar as they may help to explain the dysphoria associated with transgressing self-imposed dietary rules [16, 41, 50]. Although inconsistencies may be due to methodological problems (e.g., the use of instruments whose validity is debated), the current agreement that these aspects may be related encourages prospective longitudinal research to identify causal psychological risk factors. Also addressed in Criterion B, dietary extremism present in ON may result in nutritional deficiencies and medical complications similar to those of other F&EDs [50–52]. However, longitudinal data to support this hypothesis are not available and further work in this area is needed.

The Criterion B statement with the lowest agreement (85.2%) – although still meeting the consensus- threshold – was statement 11, referring to both low body weight and sociocultural ideals. This suggests that the mixed results in the literature are reflected in what the panel is observing in clinical practice. Although weight loss might be expected in ON due to avoidance of numerous food categories (similar to what is observed in patients with AN), published research has been inconclusive or revealed the opposite trend, demonstrating a correlation between higher BMI and more ON symptomatology [34, 64, 65].

Also related to Criterion B statement 11 is the potential role of culture that must be taken into account when defining ON, particularly the sociocultural health ideals heavily present in Western countries [22, 53–55] and geo-socio-cultural prohibitions of certain foods in different populations [57, 58]. Individual perception of healthy eating is inextricably linked with social contexts [59] and can be also reflected as a public health goal by governments and medical authorities [60]. It is important to note that the current study’s expert panel did not include participants from many areas of the world, and this does not mean that ON does not exist in those areas. Studies of the relationship between ON and quality of life in China did not show the same moderate-strong correlations with disordered eating and other mental health outcomes reported in Western samples [54, 56]. The dysfunctional eating behaviors commonly accompanying ON are frequently approved and celebrated in Western cultures [61], where the “healthy beauty ideal” emphasizes individual responsibility for ingesting the “correct” foods while adhering to dominant esthetic standards [62]. In recent years, the spread of strict sociocultural ideas of beauty may have exacerbated deviant eating patterns in vulnerable individuals, leading for example to the pathologic preoccupation with muscularity and leanness as a result of desiring the perfect body, which characterizes muscle dysmorphophobia (MD) [63]. This disorder implies the presence of disturbing and extreme attitude and desire to gain body mass in an effort to achieve the muscular ideal prominently promoted by western societies [64]. An important issue to resolve for future studies is examining the relationship between MD and ED symptoms, as well as between MD and orthorexic tendencies especially considering that this disease can have important implications for eating habits and the treatment of feeding disturbances [65].

The limited data on ON from Asian countries is mixed. In one sample of elderly Chinese people, He and colleagues failed to find the expected correlations between ON and measures of psychological ill-health, and reported small positive associations with wellbeing [22]. However, in two younger Chinese samples of adolescents [66] and college undergraduates [54], ON symptoms were modestly correlated with measures of disordered eating and cognitive restraint, and in the undergraduate sample, strongly correlated with maladaptive inflexible eating attitudes. Given frequent generational changes in knowledge, beliefs, and values around health and healthy eating, cohort effects may be associated with age differences in the correlates, and even features, of ON. In view of these aspects, culturally-sensitive diagnostic criteria for ON should be considered.

## Limitations

The proposed preliminary criteria for Orthorexia Nervosa presented here are robust, highly agreed-upon statements based on the experience and expertise of an international panel of researchers and treatment professionals with strong publication records and varied disciplinary backgrounds. However although the panel included wide and different perspectives, it must be acknowledged that many countries and geographical regions, particularly Central and South America, the Caribbean, Africa and Asia, the South Pacific, and Eastern Europe were either not represented or represented by far fewer participants than Western Europe and the United States. Expansion of ON criteria would benefit from additional recruitment of experts in a wider geographical range.

Another limitation of the current study is simply that it is based on expert opinion rather than experimental or empirical evidence, case studies, or patient report. This is indeed the purpose of the consensus-building process, and the Delphi methodology was used to ensure that decisions were made with the highest level of rigor; nevertheless, future research will be needed to verify the validity of these criteria in relation to the individual experience of ON.

## Conclusion

This paper provides the first-ever consensus-built proposed diagnostic criteria and standard definition for Orthorexia Nervosa reflecting high agreement among international, multidisciplinary experts. It provides a crucial starting point for future studies of ON that can shed light on the prevalence, risk factors and pathophysiology of this condition and lead the way toward better identification and treatment modalities.

What is already known on this subject?

Although several attempts have been made by individual authors to propose a series of diagnostic criteria for Orthorexia Nervosa, no standardized definition has been agreed upon or included in any international disease classification. This has resulted in theoretical publications that do not reflect what professionals are observing in clinical practice, a lack of empirical primary evidence, and difficulty advancing research identifying risk and protective factors, pathophysiology, functional consequences, and evidence-based therapeutic approaches.

What this study adds?

A final list of 27 statements concerning ON that can be used as agreed-upon criteria for screening research participants, evaluating treatment protocols, and expanding prevalence data between groups.

## Supplementary Information

Below is the link to the electronic supplementary material.

Supplementary file1 (DOCX 132 KB)

Supplementary file2 (DOCX 133 KB)
