# Correction: A consensus document on definition and diagnostic criteria for orthorexia nervosa

**DOI:** 10.1007/s40519-023-01599-4

**Published:** 2023-09-16

**Authors:** Lorenzo M. Donini, Juan Ramón Barrada, Friederike Barthels, Thomas M. Dunn, Camille Babeau, Anna Brytek-Matera, Hellas Cena, Silvia Cerolini, Hye-hyun Cho, Maria Coimbra, Massimo Cuzzolaro, Claudia Ferreira, Valeria Galfano, Maria G. Grammatikopoulou, Souheil Hallit, Linn Håman, Phillipa Hay, Masahito Jimbo, Clotilde Lasson, Eva-Carin Lindgren, Renee McGregor, Marianna Minnetti, Edoardo Mocini, Sahar Obeid, Crystal D. Oberle, Maria-Dolores Onieva-Zafra, Marie-Christine Opitz, María-Laura Parra-Fernández, Reinhard Pietrowsky, Natalija Plasonja, Eleonora Poggiogalle, Adrien Rigó, Rachel F. Rodgers, Maria Roncero, Carmina Saldaña, Cristina Segura-Garcia, Jessica Setnick, Ji-Yeon Shin, Grazia Spitoni, Jana Strahler, Nanette Stroebele-Benschop, Patrizia Todisco, Mariacarolina Vacca, Martina Valente, Màrta Varga, Andrea Zagaria, Hana Flynn Zickgraf, Rebecca C. Reynolds, Caterina Lombardo

**Affiliations:** 1 Sapienza University, Rome, Italy; 2 Universidad de Zaragoza, Saragossa, Spain; 3 Heinrich Heine University Düsseldorf, Düsseldorf, Germany; 4 University of Northern Colorado, Greeley, USA; 5 Ecole de Psychologues Praticiens, Lyon, France; 6 University of Wroclaw, Wroclaw, Poland; 7 University of Pavia, Pavia, Italy; 8 Chung-Ang University, Seoul, South Korea; 9 University of Coimbra, CINEICC, Coimbra, Portugal; 10 University of Coimbra, Coimbra, Portugal; 11 University of Thessaly, Larissa, Greece; 12 Holy Spirit University of Kaslik, Jounieh, Lebanon; 13 Halmstad University, Halmstad, Sweden; 14 Western Sydney University, Sydney, Australia; 15 University of Michigan, Ann Arbor, USA; 16 University of Toulouse-Jean Jaures, Toulouse, France; 17 The EN:SPIRE Clinic, Bengaluru, India; 18 Lebanese American University, Beirut, Lebanon; 19 Texas State University, San Marcos, USA; 20 University of Castilla-La Mancha, Ciudad Real, Spain; 21 The University of Edinburgh, Edinburgh, UK; 22 University of Bordeaux, Bordeaux, France; 23 Institute of Psychology, ELTE Eötvös Loránd University, Budapest, Hungary; 24 Northeastern University, Boston, USA; 25 University of Valencia, Valencia, Spain; 26 Universitat de Barcelona, Barcelona, Spain; 27 University of Catanzaro, Catanzaro, Italy; 28 International Federation of Eating Disorder Dietitians, Dallas, USA; 29 University of Freiburg, Freiburg, Germany; 30 University of Hohenheim, Stuttgart, Germany; 31 Casa Di Cura Villa Margherita, Vicenza, Italy; 32 Amedeo Avogadro University of Eastern Piedmont, Vercelli, Italy; 33 Semmelweis University, Budapest, Hungary; 34 Children’s Healthcare of Atlanta, Emory University, Atlanta, USA; 35 ICS MAUGERI IRCCS, Pavia, Italy; 36 Effat University (KSA), Jeddah, Saudi Arabia; 37 Lapeyronie Hospital, CHRU Montpellier, Montpellier, France; 38 School of Population Health, UNSW Sydney, Sydney, NSW 2052 Australia

**Correction: Eating and Weight Disorders—Studies on Anorexia, Bulimia and Obesity (2022) 27:3695–3711** **https://doi.org/10.1007/s40519-022-01512-5**

In this article Rebecca C. Reynolds was missing from the author list. The complete correct author group is given below.

Lorenzo M. Donini, Juan Ramón Barrada, Friederike Barthels, Thomas M. Dunn, Camille Babeau, Anna Brytek-Matera, Hellas Cena, Silvia Cerolini, Hye-hyun Cho, Maria Coimbra, Massimo Cuzzolaro, Claudia Ferreira, Valeria Galfano, Maria G. Grammatikopoulou, Souheil Hallit, Linn Håman, Phillipa Hay, Masahito Jimbo, Clotilde Lasson, Eva-Carin Lindgren, Renee McGregor, Marianna Minnetti, Edoardo Mocini, Sahar Obeid, Crystal D. Oberle, Maria-Dolores Onieva-Zafra, Marie-Christine Opitz, María-Laura Parra-Fernández, Reinhard Pietrowsky, Natalija Plasonja, Eleonora Poggiogalle, Adrien Rigó, Rachel F. Rodgers, Maria Roncero, Carmina Saldaña, Cristina Segura-Garcia, Jessica Setnick, Ji-Yeon Shin, Grazia Spitoni, Jana Strahler, Nanette Stroebele-Benschop, Patrizia Todisco, Mariacarolina Vacca, Martina Valente, Màrta Varga, Andrea Zagaria, Hana Flynn Zickgraf, Rebecca C. Reynolds & Caterina Lombardo.

The original article [1] has been corrected.
