# Supplementary material for: A consensus document on definition and diagnostic criteria for orthorexia nervosa
Source: Eat Weight Disord. 2022 Nov 27;27(8):3695–711. doi: 10.1007/s40519-022-01512-5 (PMC9803763; doi:10.1007/s40519-022-01512-5)
Supplement: Supplementary file 1 — Supplementary file1 (DOCX 132 KB) [file 40519_2022_1512_MOESM1_ESM.docx]

**DEFINITION of ORTHOREXIA NERVOSA – ROUND 2**

**CRITERION A: DEFINITION, CLINICAL ASPECTS AND DURATION**

| **Mandatory criteria for the definition of ON** | **agree** | **disagree** | **Defence** |
| --- | --- | --- | --- |
| ON is a **mental health disorder** associated with distress, impairment, and reduced wellbeing and falling within the DSM-5 category of “Feeding and Eating Disorders”. |  |  |  |
| The definition of “**healthful eating**” or “pure eating” includes a dietary theory or set of beliefs whose specific details may vary. ON often refers to “healthy" food as pure, clean, organic, right, correct, natural, safe; “unhealthy” food is often referred to as processed, with added ingredients, prepared, treated, toxic, contaminated such as to represent harmful consequences for the individual’s health. It might also include any other definition of healthy or unhealthy according to the affected individual (his/her background/culture/knowledge/moment in life) or to dietary trends and cultures. |  |  |  |
| ON is characterized by a strong preoccupation with one's eating behavior and with a self-imposed **rigid and inflexible rules** which may be strictly controlled and include spending an excessive amount of time for planning, obtaining, preparing and/or eating one’s food. |  |  |  |
| ON-related behavior involves disturbances of eating habit that may negatively affect **health status**, and quality of life. |  |  |  |

**Aspects that are frequently present in subjects with ON**

| Individuals with ON experience **emotional distress**, anxiety (if they are confronted with food, they believe to be unhealthy and they fear they might be impaired by eating them), problems concerning attention and concentration (if an individual think about healthy eating all day) and a feeling of guilt as a consequence of not being able to eat healthy. |  |  |  |
| --- | --- | --- | --- |
| **Self-esteem** in ON individuals seems to depend on the adherence to dietary rules. |  |  |  |
| Individuals with ON are characterized by a moral/spiritual component leading to a **feeling of ethical superiority** over the lifestyle and eating habits of others that becomes part of their belief system and identity. |  |  |  |
| In ON the observance of **rigid food rules** is maintained despite consequences on health, relationships, social life and quality of life. |  |  |  |
| ON includes **emotional** (e.g. feeling guilty after having eating food considered to be unhealthy), **cognitive** (e.g. problems concern attention and concentration) and/or **social** (e.g. social exclusion) consequences, that have a negative effect on the individuals educational, work or social life. |  |  |  |

Should other aspects be considered in the definition of orthorexia nervosa?

If yes, please indicate which parameter you propose to consider and why.

**DURATION**

|  | **agree** | **disagree** | **Defence** |
| --- | --- | --- | --- |
| Symptoms should last at least **6 months**. However, if there is a severe impairment of health (e.g. severe malnutrition), the diagnosis can be given even after 3 months. |  |  |  |

**CRITERION B: CONSEQUENCES**

|  | **agree** | **disagree** | **Defence** |
| --- | --- | --- | --- |
| As a result of the excessive amount of time devoted to their diet (reading about, acquiring and/or preparing foods), individuals with ON **miss time or activities** in their personal, vocational, and/or academic lives. |  |  |  |
| ON may result in **social isolation** and modification of social relationships. |  |  |  |
| The food selectivity, that characterises ON individuals, can contribute to cause **nutritional deficiencies**, such as anaemia, extreme weight loss, global or selective malnutrition and damage to health. |  |  |  |
| The rigid eating rules may result in low body weight and sometimes the sociocultural ideals of healthiness, at least in Western countries, may overlap greatly with thin and muscular ideals. However this **low weight** may be better conceived as a **side effect** or a consequence of ON instead of as the result of body dissatisfaction. |  |  |  |

Should other aspects be considered in the definition of orthorexia nervosa?

If yes, please indicate which parameter you propose to consider and why.

**CRITERION C: ONSET of ON**

|  | **agree** | **disagree** | **Defence** |
| --- | --- | --- | --- |
| ON seems to be associated with the development of other forms of EDs and/or with migration to other forms of EDs.  It may precede others EDs, coexist with EDs, follow other EDs (representing in this case a faulty coping strategy when no longer able to practice other ED behaviors).  It might serve as a coping strategy for individuals affected with AN to continue restricting their diet. |  |  |  |

Should other aspects be considered in the onset of Orthorexia Nervosa?

If yes, please indicate which parameter you propose to consider and why.

**CRITERION D: EXCLUSION CRITERIA**

|  | **agree** | **disagree** | **Defence** |
| --- | --- | --- | --- |
| The **food selection and/or exclusion** from the diet is not attributable to the necessity to threat specific clinical conditions (e.g. renal insufficiency, obesity, food allergies and intolerances). |  |  |  |
| The food selection and/or exclusion from the diet is not better explained by another mental disorder (e.g. health anxiety, OCD, anorexia nervosa, bulimia nervosa, psychotic disturbances, somatoform disorders). |  |  |  |
| The food selection and/or exclusion from the diet is not attributable to economic conditions, values, cultural, religious beliefs or delirious ideas. |  |  |  |

Should other aspects be considered as exclusion criteria of Orthorexia Nervosa?

If yes, please indicate which parameter you propose to consider and why.

**OTHER CHARACTERISTICS ASSOCIATED OR POSSIBLY RISK FACTORS**

**Although evidence is not consistent, ON seems to be associated with:**

|  | **agree** | **disagree** | **Defence** |
| --- | --- | --- | --- |
| Higher level of education and socio-economic status. |  |  |  |
| A specific age or gender. |  |  |  |
| Body weight concerns or variations during life, diet influenced by others or restriction of calories intake. |  |  |  |
| Physical shape or body image disturbances, internalization of Western ideals of thinness or muscularity . |  |  |  |
| Impulsivity and appearance anxiety |  |  |  |

**A higher prevalence of ON is associated with:**

| Competitive sports, athletic performance concerns and high physical exercise frequency |  |  |  |
| --- | --- | --- | --- |
| History of others EDs or mental disorders (e.g. OCD) |  |  |  |
| (Psycho) somatic problems, hypochondria, depressive symptoms, anxiety (generalized or specific) |  |  |  |
| Perfectionism, need of control, low self-esteem, narcissism, self-criticism and tendency to impose excessively high standards for oneself |  |  |  |
| Being excessively influenced by media, social networks, online platforms and websites related to eating behaviours and/or physical appearance |  |  |  |
| Alcohol and drug addiction |  |  |  |
| Vegan, vegetarian eating habits |  |  |  |
| Emotional dysregulation |  |  |  |
| University and professional choices (e.g. dietician, nutritionist) |  |  |  |

Should other characteristics associated with orthorexia nervosa be considered?

If yes, please indicate which conditions you propose to consider and why.

**DIFFERENTIAL DIAGNOSIS with other psychiatric diseases**

|  | **agree** | **disagree** | **Defence** |
| --- | --- | --- | --- |
| The fundamental differences with **AN** are that:   - in ON, appearance concerns are not central, physical appearance is not overvalued and there is no spasmodic search for thinness - in AN the goal is to lose weight/ maintain current weight while in ON the main goal is to be as healthy as possible - in AN self-esteem revolves around weight/shape while in ON self-esteem revolves around the ability to follow the self-imposed dietary rules |  |  |  |
| The fundamental differences with **OCD** are that in ON obsessions and compulsions only concern eating behavior and health.  Moreover individuals with OCD experience ego-dystonic obsessions and try to ignore or suppress those unwanted thoughts and urges, whereas individuals with ON experience ego-syntonic obsessions about food/eating that are considered appropriate and should not be ignored. |  |  |  |
| In individuals with **ARFID**, food restriction is the result of one of three subtypes: 1) An aversive experience with food causing a conditioned negative response to eating, such as choking 2) Apparent lack of interest in eating 3) Highly selective eating based on the sensory properties of food, such as color, taste, or texture. On the contrary, in  individuals with ON food restriction is the result of worries about the healthiness of a certain food. |  |  |  |

Should other aspects be considered in the differential diagnosis with orthorexia nervosa?

If yes, please indicate which risk factor you propose to consider and why.

**Legend:** ON = orthorexia nervosa; AN = anorexia nervosa; OCD = Obsessive–compulsive disorder; ARFID = avoidant restrictive food intake disorder.
