# Supplementary material for: A consensus document on definition and diagnostic criteria for orthorexia nervosa
Source: Eat Weight Disord. 2022 Nov 27;27(8):3695–711. doi: 10.1007/s40519-022-01512-5 (PMC9803763; doi:10.1007/s40519-022-01512-5)
Supplement: Supplementary file 2 — Supplementary file2 (DOCX 133 KB) [file 40519_2022_1512_MOESM2_ESM.docx]

**DEFINITION of ORTHOREXIA NERVOSA – ROUND 1**Please give your opinion on the following aspects concerning only the **DEFINITION** of orthorexia nervosa:

**CRITERION A: DEFINITION, CLINICAL ASPECTS AND DURATION**

|  | **agree** | **disagree** | **Defence** |
| --- | --- | --- | --- |
| ON is a distinct mental health disorder category and not just a subset of another disorder |  |  |  |
| ON is a distinct mental health disorder and it is not merely a behavioural or lifestyle phenomenon |  |  |  |
| Individuals affected by ON adopt a self-imposed rigid diet, strictly controlled |  |  |  |
| Individuals affected by ON adopt a diet characterized by a ritualized pattern of eating and selective avoidance of food |  |  |  |
| ON is characterized by rigid avoidance of food believed to be unhealthy or not pure |  |  |  |
| ON often refers “Healthy" food as pure, organic, proper, correct, natural, safe; “unhealthy” food is often referred to as polluted, chemical, impure or obtained through unhealthy production methods |  |  |  |

|  | **agree** | **disagree** | **Defence** |
| --- | --- | --- | --- |
| The definition should include “healthful eating” or “pure  eating” defined as a dietary theory or set of beliefs  whose specific details may vary. This theory can be based on generally accepted nutritional theories, but it can also be a unique / individual definition of healthy eating of the affected person. |  |  |  |
| ON is characterized by long-term planning for food |  |  |  |
| ON is characterized by lack of flexibility in diet |  |  |  |
| ON is characterized by overrated ideas about the positive or negative consequences of foods on health |  |  |  |
| ON is characterized by the desire to improve or preserve health status by healthy eating |  |  |  |
| ON is characterized by the desire to avoid the negative consequences of unhealthy foods or eating patterns |  |  |  |
| ON is characterized by an obsessive preoccupation with a diet believed to be healthy. Obsession refers to the presence of persistent, intrusive thoughts directed to healthy foods and healthy eating. |  |  |  |
| Since ON involves disturbance of eating habits that negatively affect health status, it should be classified as an ED |  |  |  |
| ON is characterized by body dissatisfaction |  |  |  |

Should other aspects be considered in the definition of Orthorexia Nervosa?

If yes, please indicate which parameter you propose to consider and why.

**DURATION**

|  | **agree** | **disagree** | **Defence** |
| --- | --- | --- | --- |
| Symptoms should persist at least 3 months |  |  |  |
| Symptoms should persist at least 6 months |  |  |  |

**CRITERION B: CONSEQUENCES**

|  | **agree** | **disagree** | **Defence** |
| --- | --- | --- | --- |
| The food selectivity, based on personal beliefs, can contribute to cause nutritional deficiencies, malnutrition and damage to health |  |  |  |
| ON may lead to being underweight, however weight loss is not intentional but an indirect consequence |  |  |  |
| Individuals with ON experience emotional distress as a consequence of not being able to eat healthy |  |  |  |
| Individuals with ON feel guilty or self-loathing as a consequence of transgressing the healthy diet |  |  |  |
| ON may result in social isolation and modification of social relationships |  |  |  |
| Individuals with ON exhibit feelings of superiority over the lifestyle and eating habits of others |  |  |  |
| Self-esteem in ON seems to depend on the adherence to dietary rules. |  |  |  |
| The fixation on healthy eating is characterized by spending excessive amounts of money relative to one's income on foods because of their perceived quality and composition. |  |  |  |
| The fixation on healthy eating is characterized by spending a considerable amount of time reading about, acquiring and/or preparing specific types of foods based on their perceived quality and composition. |  |  |  |

Should other aspects be considered in the definition of orthorexia nervosa?

If yes, please indicate which parameter you propose to consider and why.

**CRITERION C: ON ONSET**

|  | **agree** | **disagree** | **Defence** |
| --- | --- | --- | --- |
| ON may precede other EDs |  |  |  |
| ON may characterize the recovery phase of other EDs |  |  |  |

Should other aspects be considered in the onset of Orthorexia Nervosa?

If yes, please indicate which parameter you propose to consider and why.

**CRITERION D: EXCLUSION CRITERIA**

|  | **agree** | **disagree** | **Defence** |
| --- | --- | --- | --- |
| The food selection and/or exclusion from the diet is not attributable to the intention of losing weight (e.g. overweight subjects), to concurrent medical conditions (e.g. food allergies and intolerances). |  |  |  |
| The food selection and/or exclusion from the diet is not better explained by another mental disorder. |  |  |  |
| The food selection and/or exclusion from the diet is not attributable to economic conditions, values, cultural or religious beliefs. |  |  |  |

Should other aspects be considered as exclusion criteria of Orthorexia Nervosa?

If yes, please indicate which parameter you propose to consider and why.

**OTHER CHARACTERISTICS ASSOCIATED OR POSSIBLY RISK FACTORS**

**A higher prevalence of ON is associated with:**

|  | **agree** | **disagree** | **Defence** |
| --- | --- | --- | --- |
| Higher level of education and socio-economic status |  |  |  |
| Younger age |  |  |  |
| Female sex |  |  |  |
| Following a diet imposed by others (ON by proxy) |  |  |  |
| Body weight concerns |  |  |  |
| Obesity |  |  |  |
| Conspicuous weight variations during life |  |  |  |
| Restriction of calories intake and/or adoption of a low-calorie diet |  |  |  |
| History of others ED or mental disorders |  |  |  |
| Physical shape preoccupation |  |  |  |
| Body image disturbances |  |  |  |
| Competitive sports, athletic performance concerns and high physical exercise frequency |  |  |  |
| Internalization of Western ideals of thinness or muscularity |  |  |  |
| Being excessively influenced by media, social networks, online platforms and websites related to eating behaviours and/or physical appearance |  |  |  |
| Necessity to define one's identity through food choices |  |  |  |
| Desire to be part of a certain community |  |  |  |
| Perfectionism, narcissism, and tendency to impose excessively high standards for oneself |  |  |  |
| Impulsivity and appearance anxiety |  |  |  |
| (Psycho) somatic problems and hypochondria |  |  |  |
| Need of control |  |  |  |
| Depressive symptoms |  |  |  |
| Anxieties, generalized or specific |  |  |  |

**A lower prevalence of ON is associated with:**

|  | **Agree** | **Disagree** | **Defence** |
| --- | --- | --- | --- |
| Divorce |  |  |  |
| Free-lance professions |  |  |  |
| Smoking habits |  |  |  |

**Other aspects associated or not to ON:**

|  | **Agree** | **Disagree** | **Defence** |
| --- | --- | --- | --- |
| University and professional choices may be influenced by pre-existing ON and viceversa |  |  |  |
| No correlation with ON and self-esteem |  |  |  |

Should other characteristics associated with orthorexia nervosa be considered ?

If yes, please indicate which conditions you propose to consider and why.

**DIFFERENTIAL DIAGNOSIS with other psychiatric diseases**

Please give your opinion on the following aspects concerning only the **DIFFERENTIAL DIAGNOSIS** with other psychiatric diseases:

|  | **agree** | **disagree** | **Defence** |
| --- | --- | --- | --- |
| The fundamental difference with AN is that  in ON, there is no fear of gaining weight or becoming fat |  |  |  |
| ON might serve as a coping strategy for individuals affected with AN |  |  |  |
| The fundamental difference with OCD is that in ON, obsessions and compulsions only concern eating behaviour |  |  |  |
| Another difference is that OCD is egodystonic, while ON is egosynthonic |  |  |  |
| Another difference is that the obsession in individuals with OCD are experienced with urge, while this doesn’t occur in individuals affected by ON |  |  |  |
| In individuals with ARFID the food avoidance or restriction associated with insufficient intake most commonly develops in infancy or early childhood |  |  |  |
| Individuals with ARFID may present a lack of interest in eating or food |  |  |  |
| ARFID has a higher prevalence in males while ON has a higher prevalence in females |  |  |  |
| While individuals with ARFID worry about taste, smell or texture of specific foods, individuals affected with ON worry about the healthiness of the food. |  |  |  |
| ON seems to be associated with migration to other forms of EDs |  |  |  |

Should other aspects be considered in the differential diagnosis with orthorexia nervosa?

If yes, please indicate which risk factor you propose to consider and why.

Legend: ON = orthorexia nervosa; AN = anorexia nervosa; OCD = Obsessive–compulsive disorder; ASD = Autism Spectrum Disorder; ARFID = avoidant restrictive food intake disorder;
